# 

**Published:** 1860-07

**Authors:** 


### RECEIPTS FOR THE JOURNAL, FOR JUNE 1860.

| | | |
|---|---|---|
| S Wickersham, Ill | Vol 3 | 2 00 |
| J Walker Ill | " 1 | 2 00 |
| M Thornell, Mo | " 3 | 2 00 |
| C S Tucker, Mich | " 3 | 2 00 |
| John Gregory, Ill | " 3 | 2 00 |
| K I Rich, Ill | Vol 3 | 2 00 |
| J G Harlan, Iowa | " 3 | 2 00 |
| E Keith, Iowa | " 3 | 2 00 |
| J S Collings, Ind | " 3 | 2 00 |

